# *Gdf11* gene transfer prevents high fat diet-induced obesity and improves metabolic homeostasis in obese and STZ-induced diabetic mice

**DOI:** 10.1186/s12967-019-02166-1

**Published:** 2019-12-17

**Authors:** Bingxin Lu, Jianing Zhong, Jianfei Pan, Xiaopeng Yuan, Mingzhi Ren, Liping Jiang, Yuqing Yang, Guisheng Zhang, Dexi Liu, Chunbo Zhang

**Affiliations:** 1 School of Pharmacy, Nanchang University, Nanchang, 330031 Jiangxi China; 2 Provincial Key Laboratory for Drug Targeting and Drug Screening Research, Nanchang, 330031 Jiangxi China; 3 The Science Research Center, Gannan Medical University, Ganzhou, 341000 Jiangxi China; 4 Department of Pharmaceutical and Biomedical Sciences, University of Georgia College of Pharmacy, Athens, GA 30602 USA

**Keywords:** GDF11, Insulin resistance, Glucose homeostasis, Fatty liver, Hydrodynamic gene transfer

## Abstract

**Background:**

The growth differentiation factor 11 (GDF11) was shown to reverse age-related hypertrophy on cardiomyocytes and considered as anti-aging rejuvenation factor. The role of GDF11 in regulating metabolic homeostasis is unclear. In this study, we investigated the functions of GDF11 in regulating metabolic homeostasis and energy balance.

**Methods:**

Using a hydrodynamic injection approach, plasmids carrying a mouse *Gdf11* gene were delivered into mice and generated the sustained *Gdf11* expression in the liver and its protein level in the blood. High fat diet (HFD)-induced obesity was employed to examine the impacts of *Gdf11* gene transfer on HFD-induced adiposity, hyperglycemia, insulin resistance, and hepatic lipid accumulation. The impacts of GDF11 on metabolic homeostasis of obese and diabetic mice were examined using HFD-induced obese and STZ-induced diabetic models.

**Results:**

*Gdf11* gene transfer alleviates HFD-induced obesity, hyperglycemia, insulin resistance, and fatty liver development. In obese and STZ-induced diabetic mice, *Gdf11* gene transfer restores glucose metabolism and improves insulin resistance. Mechanism study reveals that *Gdf11* gene transfer increases the energy expenditure of mice, upregulates the expression of genes responsible for thermoregulation in brown adipose tissue, downregulates the expression of inflammatory genes in white adipose tissue and those involved in hepatic lipid and glucose metabolism. Overexpression of GDF11 also activates TGF-β/Smad2, PI3K/AKT/FoxO1, and AMPK signaling pathways in white adipose tissue.

**Conclusions:**

These results demonstrate that GDF11 plays an important role in regulating metabolic homeostasis and energy balance and could be a target for pharmacological intervention to treat metabolic disease.

## Introduction

Growth differentiation factor 11 (GDF11), also known as bone morphogenetic protein 11, is a member of the TGF-β family playing pleiotropic roles in mammalian development [1]. GDF11 is expressed in multiple tissues, including heart, kidney, skeletal muscle, nervous system, olfactory system, retina, pancreas, intestine. GDF11 has been considered as a rejuvenation factor capable of reversing aging-related dysfunctions in multiple organs including cardiac hypertrophy, skeletal muscle dysfunction, and cerebral vasculature dysfunction [1–5] although conflict results have been reported [6–9]. The correlation of blood circulation level of GDF11 with aging was conflicting in humans showing increase, decrease, or no change with aging [6, 10–14]. Some reports showed that the circulation level of GDF11 increased in type 2 diabetes (T2D) or obesity in humans and mice [15, 16]. However, some reports are contrary, showing no change with type 2 diabetes and obesity in humans [13, 14].

More recent studies have suggested the potential of GDF11 for the treatment of metabolic diseases such as atherosclerosis, type 2 diabetes, and diabetes-related vascular dysfunctions [15, 17, 18]. Administration of recombinant GDF11 protein (rGDF11) improved β cell function and attenuated the symptom of T2D in diabetic mice [17]. Mei and colleagues demonstrated a reduction of atherosclerotic plaques and improvement of endothelial injury using AAV-mediated *Gdf11* gene transfer in *ApoE*^−*/*−^ mice [15]. Employing the psoriasis-like skin inflammation model and high-fat diet (HFD) induced obese mice, Wang and colleagues demonstrated the anti-inflammatory activity of GDF11 [15, 19]. Also, GDF11 activated multiple signal pathways, such as Smad, Akt, and p38 MAPK, which were involved in the development of obesity, fatty liver, and insulin resistant [1, 20–22], implying that GDF11 may play an important role in obesity and obesity-related metabolic disorders. However, little is known about the function of GDF11 in the development of obesity and obesity-related metabolic disorders.

The focus of the current study is to investigate the functions of GDF11 in regulating metabolic homeostasis and energy balance in high fat diet-induced obesity mice and animals with STZ-induced diabetes. We demonstrate that GDF11 overexpression via gene transfer leads to significant improvement of metabolic homeostasis in obese mice and mice with STZ-induced diabetes, and blockade of high fat diet-induced weight gain, hyperglycemia, insulin resistance, and fatty liver development. These results support the notion that GDF11 plays a critical role in regulating metabolic homeostasis and could be considered as a therapeutic agent for the treatment of metabolic disorders.

## Materials and methods

### Materials

The pLIVE empty vector and pLIVE-SEAP plasmid (carrying secreted alkaline phosphatase gene) were purchased from Mirus Bio (Madison, WI, USA). The cDNA of the mouse *Gdf11* gene was amplified by PCR and inserted into pLIVE vector at *BamH1* and *Sac1* sites to make pLIVE-GDF11 plasmid. The new plasmid construct was amplified in *E. coli* and extracted using endotoxin-free maxi plasmid kits from Tiangen Biotech (Beijing, China). The inserted *Gdf11* gene sequence in the plasmid was verified by DNA sequencing. PrimeScript™ RT reagent kit was from Takara Bio. (Dalian, China). SYBR Green kit for real time-PCR was from Qiagen (Duesseldorf, Germany). BCA Quantitation kit for proteins was purchased from Applygen Technologies Inc. (Beijing, China). ELISA kit for GDF11 protein was from MEIMIAN (Cat. MM-44346M1, Wuhan, China). Primary antibodies against AKT (#4691), p-AKT (Thr 308, #13038), SMAD2 (#5339), p-SMAD2 (Ser 465/467, #3108), FOXO1 (#2880), AMPK (#5831), and p-AMPK (Thr172, #2535) were from Cell Signaling Technology (Danvers, CO, USA). Anti-p-FOXO1 antibody (Ser 256, ab131339) was from Abcam (Cambridge, UK). Primary antibodies against UCP1 (#23673-1-AP), UCP2 (#11081-1-AP), and β-actin (#66009-1-lg) antibodies were from Proteintech (Chicago, USA). The HRP-linked anti-mouse (Cat. ZB-2305) and anti-rabbit (Cat. ZB-2301) second antibodies were from ZSGB BIO Inc. (Beijing, China). Human insulin (Humulin) was from Eli Lilly (Indianapolis, IN, USA). Mouse Insulin ELISA kit was from Shanghai Enzyme-linked Biotechnology Co., Ltd. (Cat. ml001983, Shanghai, China). H&E staining kit was from Yulu (Cat. L11020102, Nanchang, China). The streptozotocin (STZ) was purchased from Sigma Aldrich (St. Louis, MO, USA). The high-fat diet (60% kJ/fat, 20% kJ/carbohydrate, 20% kJ/protein) and regular Chow were from Research Diets, Inc. (Cat. D12492,NJ,USA) and Keao Xieli Feed Cooperation (SPF-level mice maintaining Chow, Beijing, China), respectively.

### Animal procedure

C57BL/6 mice (male, ~ 25 g) were purchased from Charles River Laboratories China (Beijing, China). All animals were group-housed under standard conditions at 25 ± 2 °C with a 12 h light–dark cycle with free access to food and water. The animal protocol used was approved by the Animal Ethics Committee of the Nanchang University. In studies designed to examine the effect of *Gdf11* gene transfer on preventing HFD-induced obesity, mice were divided into 3 groups (5 mice each). Two groups were fed an HFD and the other with regular Chow. Each mouse was hydrodynamically injected with 25 µg (dose: 1 mg/kg) of pLIVE-GDF11 or pLIVE-SEAP control plasmid DNA, respectively, according to the previously published procedure [21]. Body weight and food intake were measured weekly. The rectal temperature of the animals was measured by an electric rectal thermometer weekly. Blood was collected at predetermined time points, and serum was prepared and stored at − 20 °C until use.

To examine the effects of *Gdf11* gene transfer on obese mice, mice were fed an HFD to establish obesity, and then divided into two groups (5 mice each). pLIVE-GDF11 or pLIVE-SEAP plasmids were hydrodynamically injected via tail vein, respectively. The volume of injected saline solution with 25 µg plasmid DNA was adjusted to 6% body weight for obese mice.

For the STZ-induced type 2 diabetic model, 15 mice were fed an HFD for 4 weeks, and injected intraperitoneally with a single dose of STZ (100 mg/kg). Blood glucose levels were measured 2 weeks later using Ultra One Touch glucosemeter (Johnson & Johnson, USA). The mice were considered diabetic when non-fasting blood glucose level was higher than 13.9 mmol/l for at least 2 consecutive days. The diabetic mice were divided into 2 groups (5 mice each) and 25 µg of plasmid DNA were hydrodynamically injected with an injection volume equal to 8% body weight. Animals were continued on an HFD for additional 4 weeks, and non-fasting blood glucose levels were determined and serum biochemistry assays were performed.

### Glucose tolerance test (GTT) and insulin tolerance test (ITT)

GTT and ITT were performed during the last week of the study on the same animals with a 2-day interval for recovery. For GTT, mice were fasted for 8 h and intraperitoneally injected with glucose in saline (0.2 g/ml, 2 g/Kg). Blood glucose was determined using glucose strips at 0, 30, 60, and 120 min after injection. For ITT, animals were fasted for 6 h and then intraperitoneally injected with insulin (100 IU/ml, 0.75 U/Kg). Blood glucose level was measured at the same time points as in GTT. Total blood was collected 2 days later for serum biochemistry. Insulin levels in the blood were determined using the Mouse Insulin ELISA Kit. Insulin resistance (HOMA-IR) was calculated using the formula as previous described [23]: HOMA-IR = (fasting insulin (ng/ml) × fasting blood glucose (mg/dl)/405).

### Histochemistry analysis

Collected tissue samples were fixed in 10% neutrally buffered formalin for at least 24 h and embedded in paraffin following the instruction of H&E staining kit. Tissue sections were made at a thickness of 5 μm followed by H&E staining. For Oil-Red O staining, freshly collected liver samples were embedded in OCT medium and frozen in liquid nitrogen. The frozen tissues were equilibrated in a cryostat and sectioned at 8 μm in thickness. The slide sections were fixed in 10% neutrally buffered formalin for 30 min, stained with 0.5% Oil Red O in 60% isopropanol for 15 min, counterstained with hematoxylin, washed three times, and then sealed in neutral gum. Histological photographs were taken under an optical microscope equipped with a Nikon camera.

### Immunohistochemistry

White adipose tissue was embedded in paraffin and cut at 5 μm thickness. The tissue sections were placed in citrate antigen repair buffer (pH 6.0) in the thermostatic water bath for antigen retrieval. Slices were then put into 3% BSA buffer and blocked at room temperature for 30 min. After blocking, the slices were incubated with primary antibody against F4/80 (dilution 1:500, Cat. GB11027, Wuhan Servicebio Technology Co., Ltd., Wuhan, China) at 4 °C overnight and HRP-linked secondary antibody (dilution 1:200, Cat. G1213, Wuhan Servicebio Technology Co., Ltd., Wuhan, China) for 50 min at room temperature. Then color development was performed with freshly prepared DAB coloring solution (Cat. G1212-200, Wuhan Servicebio Technology Co., Ltd., Wuhan, China). After counterstained nuclei with hematoxylin for 3 min, the slices were dehydrated and sealed.

### Biochemical analysis

Serum concentrations of aspartate aminotransferase (Cat. AST03, NingBo PureBio Biotechnology, Ningbo, China), alanine aminotransferase (Cat. ALT03, NingBo PureBio Biotechnology, Ningbo, China), triglycerides (E1013, Applygen Technologies Inc.), total cholesterol (E1015, Applygen Technologies Inc.), and free fatty acids (#15781, Diasy Diagnostic Systems, Frankfurt, Germany) were determined using ELISA kits. To assess the lipid contents in the liver, liver samples (100 mg) were homogenized in a mixture of chloroform and methanol (2:1, volume ratio) and incubated at 70 °C for 10 min. The homogenates were centrifuged at 2000 rpm for 5 min, and the supernatants were dried and re-dissolved in 5% Triton X-100. The amounts of cholesterol, triglyceride and free fatty acids were determined following the manufacturers’ instructions.

### Analysis of mouse energy metabolism

The individual mouse was placed in a gas-tight metabolic cage and acclimated for 1 day before the parameters of energy metabolism were determined using a TSE-PhenoMaster system (TSE Systems, Germany) as previously described [24]. The parameters monitored include water and food consumption, total animal activity, the volume of O_2_ (VO_2_) and CO_2_ (VCO_2_), and respiratory exchange ratio (RER). VO_2_ was calculated by the equation: “VO_2_” [ml/h/kg] = FlowML [ml/h] * (V1 [%^2] + V2 [%^2])/(N2Ref[%] * BodyWeight[kg] * 100.0 [%]). VCO_2_ was calculated by the equation: “VCO_2_” [ml/h/kg] = FlowML [ml/h]*dCO_2_ [%]/(BodyWeight [kg] * 100.0 [%]). RER was calculated by the equation: RER = VCO_2_ [ml/h/kg]/VO_2_ [ml/h/kg]. Energy expenditure (EE) was calculated by the equation: EE = 3.941 × VO_2_ + 1.106 × VCO_2_.

### Analysis of gene expression

Real-time PCR was performed to determine the expression level of selected genes. Total RNA was isolated from the mouse liver, white and brown adipose tissues using TRIzol reagents from Invitrogen. One μg of total RNA was reverse-transcribed to generate the first-strand cDNA using PrimeScript™ RT reagent kit. RT-PCR was performed using SYBR Green kit. GAPDH RNA served as an internal control and data were normalized using GAPDH RNA level as 1. All primer sequences employed are summarized in Additional file 1: Table S1.

### Western blotting

Proteins in brown and white adipose tissue were extracted by adipose tissue protein extraction kit (BB-312262, BestBio, Shanghai, China). Briefly, 100 mg adipose tissue was homogenized in 500 μl protein extraction buffer with protease inhibitors. The homogenate was incubated for 30 min at 4 °C and centrifuged for 15 min at 12,000*g* at 4 °C. The supernatant was collected and centrifuged one more time using the same condition. The concentration of total proteins was detected by BCA Quantitation Kits. The same amount of sample (100 µg total protein) was loaded in each well and resolved by SDS-PAGE. The protein bands after electrophoretic separation were transferred to Immobilon-P PVDF Membrane by Mini-PROTEAN^®^ Tetra system at 200 mA for 90 min with transfer buffer (25 mM Tris, 189 mM Glycine, 200 ml MeOH, 800 ml ddH_2_O). Primary antibodies (dilution 1:2000) were added and incubated overnight at 4 °C. After washing, the HRP-linked anti-mouse second antibody (dilution 1:2500) or anti-rabbit second antibody (dilution 1:2500) were added and incubated for 120 min. The specific protein bands were visualized using a Chemiluminescence imager. The relative levels of protein were scaled to the level of β-actin. The relative levels of the phosphorylated protein were normalized to the signal of their total abundance of that protein.

### Statistical analysis

Statistical analysis was performed by using the Student’s t-test, one-way ANOVA, or nonparametric test. Normality and homogeneity of variances were analyzed using Shapiro–Wilk test and Levene’s test respectively. Post-hoc comparisons were performed using LSD and SNK test in oneway ANOVA. If the data was still not a normal distribution after log transformation, Kruskal–Wallis H test and Mann–Whitney U test were performed. Results were expressed as the mean ± SEM. P < 0.05 was considered significantly different.

## Results

### Establishment of *Gdf11* overexpression in mice

**Fig. 1:**
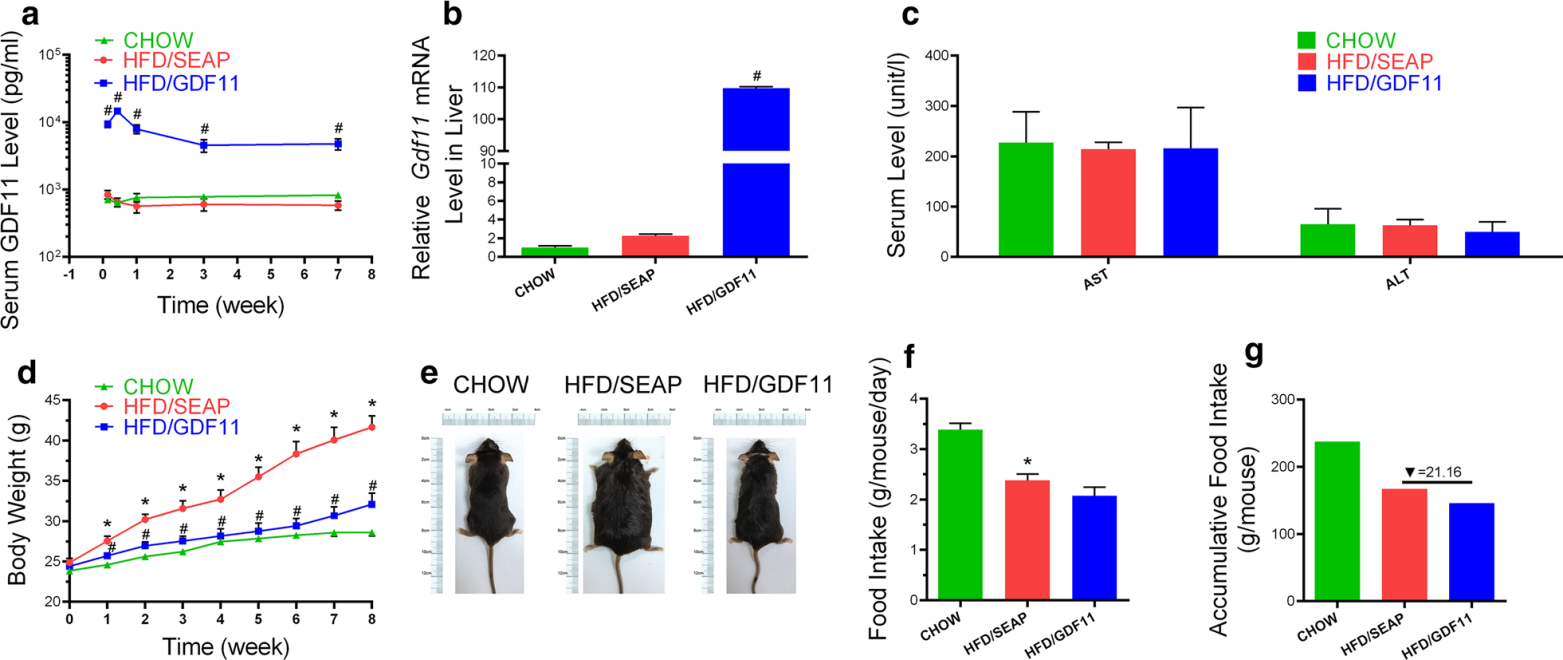
Hydrodynamic injection of *Gdf11* gene generated sustained expression of GDF11 and prevented HFD-induced weight gain. **a** Serum concentration of GDF11 as function of time post plasmid injection; **b** relative mRNA levels of *Gdf11* in the liver 8 weeks post plasmid injection; **c** serum levels of AST and ALT at the end of experiments; **d** growth curve; **e** representative images of mice at end of the experiment; **f** daily food intake; **g** accumulative food intake in 8-week period. Data represent mean ± SEM (n = 5). *P < 0.05 comparing to mice fed a Chow diet, ^#^P < 0.05 comparing to mice fed an HFD and injected with pLIVE-SEAP plasmids

Hydrodynamic tail vein injection was used to establish overexpression of *Gdf11* gene in the liver. Animals fed regular Chow food were set as the Chow control without any treatment. Mice fed HFD and hydrodynamically injected with pLIVE-SEAP plasmids (carrying secreted alkaline phosphatase gene) were set as the HFD control. The serum level of GDF11 protein was measured after hydrodynamic injection of pLIVE-GDF11 plasmids (HFD/GDF11 group). The serum concentration of GDF11 reached a peak level at about 14500 pg/ml within 48 h, and a slight decline was observed 3 days after the injection (Fig. 1a). At the end of the test, the serum concentration of GDF11 stayed at approximately 4800 pg/ml, eightfold higher than the background level at 600 pg/ml. Persistent *Gdf11* expression was confirmed by qPCR of mRNA isolated from the liver. A high level of *Gdf11* mRNA was readily detected at the end of experiments (Fig. 1b). Compared to control animals, overexpression of *Gdf11* did not cause liver damage as demonstrated by serum levels of AST and ALT (Fig. 1c).

### Impacts of pLIVE-GDF11 delivery on high-fat diet-induced weight gain and food intake

Results in Fig. 1d shows that overexpression of *Gdf11* gene prevented HFD-induced weight gain. Eight weeks after plasmid injection, control mice fed an HFD and injected with pLIVE-SEAP control plasmid showed an average body weight of 41.7 ± 1.4 g comparing to 32.1 ± 1.3 g for mice injected with pLIVE-GDF11 which is slightly higher than 28.6 ± 0.4 g of animals fed regular Chow. The size difference between treated and control animals are apparent judging by their appearance (Fig. 1e). Both accumulative and average food intake in Gdf11-treated animals fed an HFD are slightly lower than HFD-fed control mice. However, there is no statistical significance between control and pLIVE-GDF11 injected animals fed an HFD (Fig. 1f, g). One group of Chow food fed mice were also treated with GDF11 (Chow/GDF11), and no significant difference was observed in body weight change and blood glucose between Chow and Chow/GDF11 groups (Additional file 2: Figure S1).

### *Gdf11* gene transfer suppressed HFD-induced hypertrophy

**Fig. 2:**
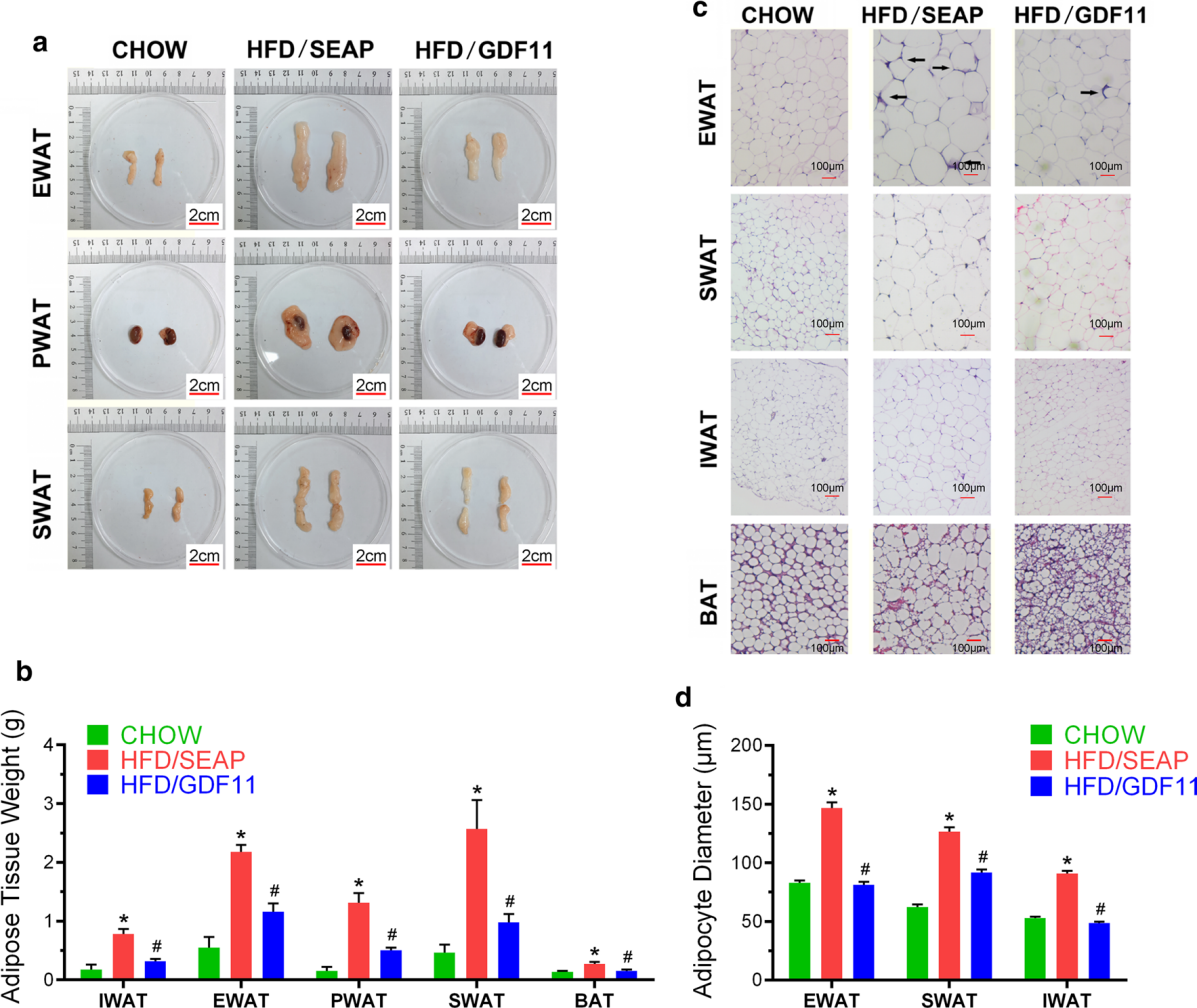
Impacts of *Gdf11* gene transfer on adipose tissues. Adipose tissues were collected at the end of 8 weeks from animals fed regular Chow or HFD with hydrodynamic injection of pLIVE-GDF11 (HFD/GDF11) or control plasmids (HFD/SEAP). **a** Representative images of adipose tissues; **b** weight of fat pads; **c** histological images of EWAT, SWAT, IWAT and BAT (×10); **d** average diameter of adipocytes. *SWAT* subcutaneous WAT; *IWAT* inguinal WAT; *PWAT* perirenal WAT; *EWAT* epididymal WAT. Data represent mean ± SEM (n = 5). Arrows point to crown-like structures and scale bars represent 2 cm in **a** and 100 µm in **c**. *P < 0.05 comparing to mice fed regular Chow, ^#^P < 0.05 comparing to HFD-fed control mice

To explore the impact of GDF11 on adipocytes, different adipose tissues were collected at the end of the experiment. Comparing to regular mice fed a regular Chow (Fig. 2a), HFD-fed control animals had significantly larger size of epididymal (EWAT), perirenal (PWAT), and part of subcutaneous (SWAT, including inguinal (IWAT), dorsolumbar subcutaneous (dlWAT), and anterior subcutaneous (asWAT)) white adipose tissues. WAT in *Gdf11*-treated animals are significantly smaller than HFD-fed control mice as confirmed by WAT weights (Fig. 2b). BATs in HFD-fed control animals weigh slightly more than those of animals either fed a regular Chow or HFD with the injection of pLIVE-GDF11 plasmids. H&E staining was performed on WAT and BAT on all animals (Fig. 2c). Average size of white adipocytes in HFD-fed control animals are significantly bigger than that of animals with *Gdf11* overexpression and animals fed a regular Chow (EWAT 146.6 ± 4.8 μm vs. 81.1 ± 2.8 μm; SWAT 126.6 ± 3.6 μm vs. 91.6 ± 2.7 μm; IWAT 90.8 ± 2.4 μm vs. 48.6 ± 1.1 μm, Fig. 2d). Crown-like structures, an indication of macrophage infiltration into WAT, were seen in EWAT of HFD-fed control animals (Fig. 2c). In BAT, HFD-fed control animals show more lipid accumulation than those of Chow-fed control and the animals injected with pLIVE-GDF11 plasmids (Fig. 2c). These results demonstrate that *Gdf11* gene transfer suppressed the HFD-induced hypertrophy.

### GDF11 improves HFD-induced glucose intolerance and insulin resistance

**Fig. 3:**
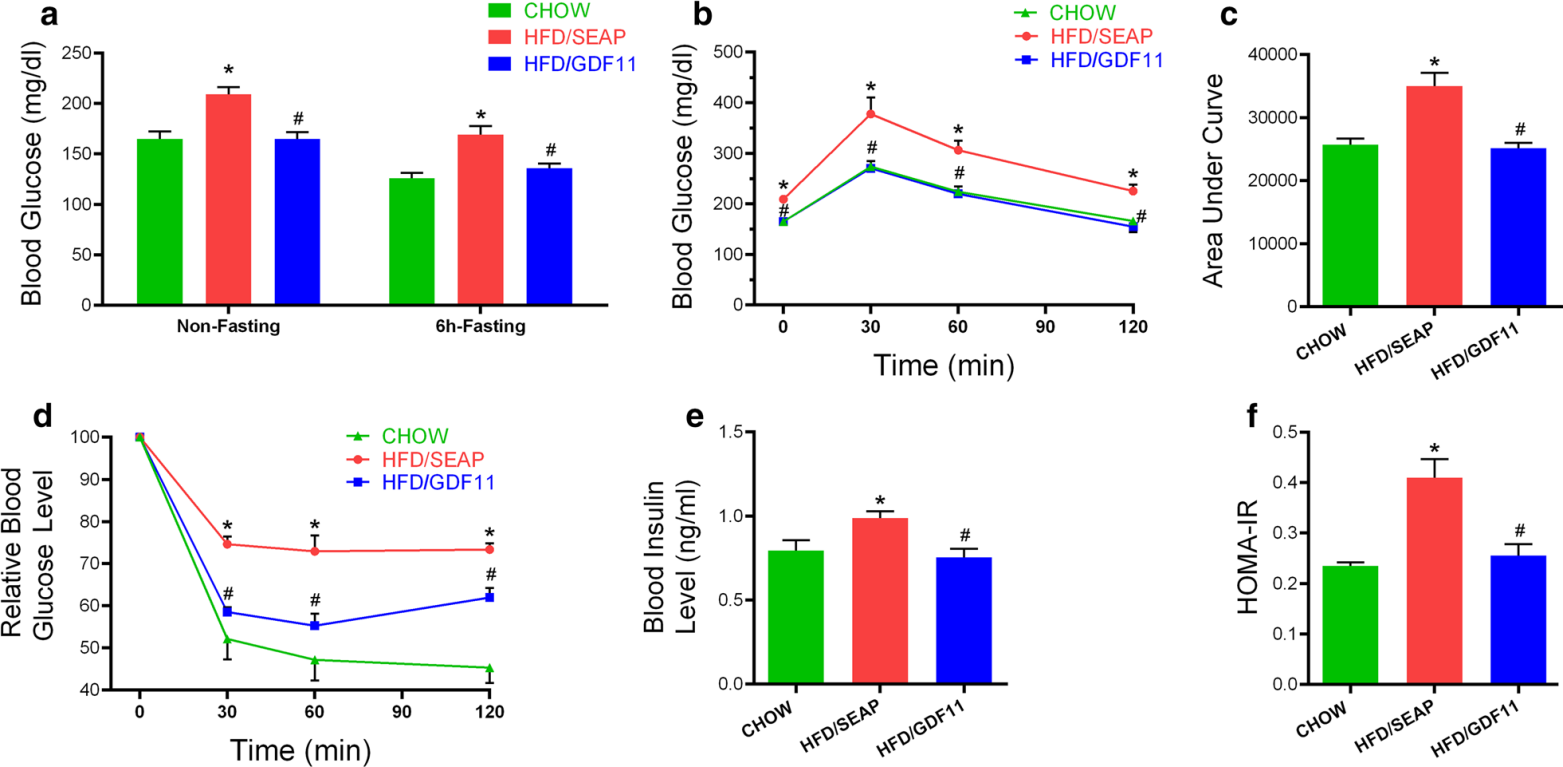
Gdf11 gene transfer suppressed HFD-induced hyperinsulinemia and maintained glucose homeostasis. **a** Blood glucose levels with or without 6 h fasting of mice at the end of the feeding period. **b** Serum glucose level of mice in GTT (2 g glucose/kg, i.p.); **c** area under the curve of GTT; **d** Relative blood glucose level of mice in ITT (0.75 U/kg, i.p.); **e** blood insulin level; **f** results of HOMA-IR analysis for insulin resistance. Data represent mean ± SEM (n = 5). *P < 0.05 comparing to mice fed regular Chow, ^#^P < 0.05 comparing to HFD-fed control animals

Obesity usually accompanies hyperglycemia, glucose intolerance and hyperinsulinemia [25]. To investigate the impact of *Gdf11* gene transfer on glucose metabolism and metabolic homeostasis, we performed GTT and ITT. Results in Fig. 3a show that mice injected with *Gdf11* gene showed a lower non-fasting and fasting glucose level comparing to that of HFD-fed control mice. GTT assay showed that GDF11-treated animals had a higher glucose clearance rate (Fig. 3b). The area under the curve (AUC) of GTT confirmed that *Gdf11* overexpression improved glucose tolerance (Fig. 3c). ITT assay showed that blood glucose levels in animals with *Gdf11* gene transfer responds to insulin injection more readily than the HFD-fed control and similar to animals fed regular Chow (Fig. 3d). Blood insulin level in pLIVE-GDF11 plasmid injected mice (0.75 ± 0.10 ng/ml) was significantly lower than that of HFD-fed control mice (0.99 ± 0.08 ng/ml) (Fig. 3e), indicating that mice treated with *Gdf11* gene were more sensitive to insulin administration. HOMA-IR confirmed that *Gdf11* gene transfer suppressed the development of insulin resistance (Fig. 3f). These results demonstrate the activity of GDF11 in suppressing HFD-induced glucose intolerance and insulin resistance.

### GDF11 blocks the development of HFD-induced fatty liver and hepatic steatosis

**Fig. 4:**
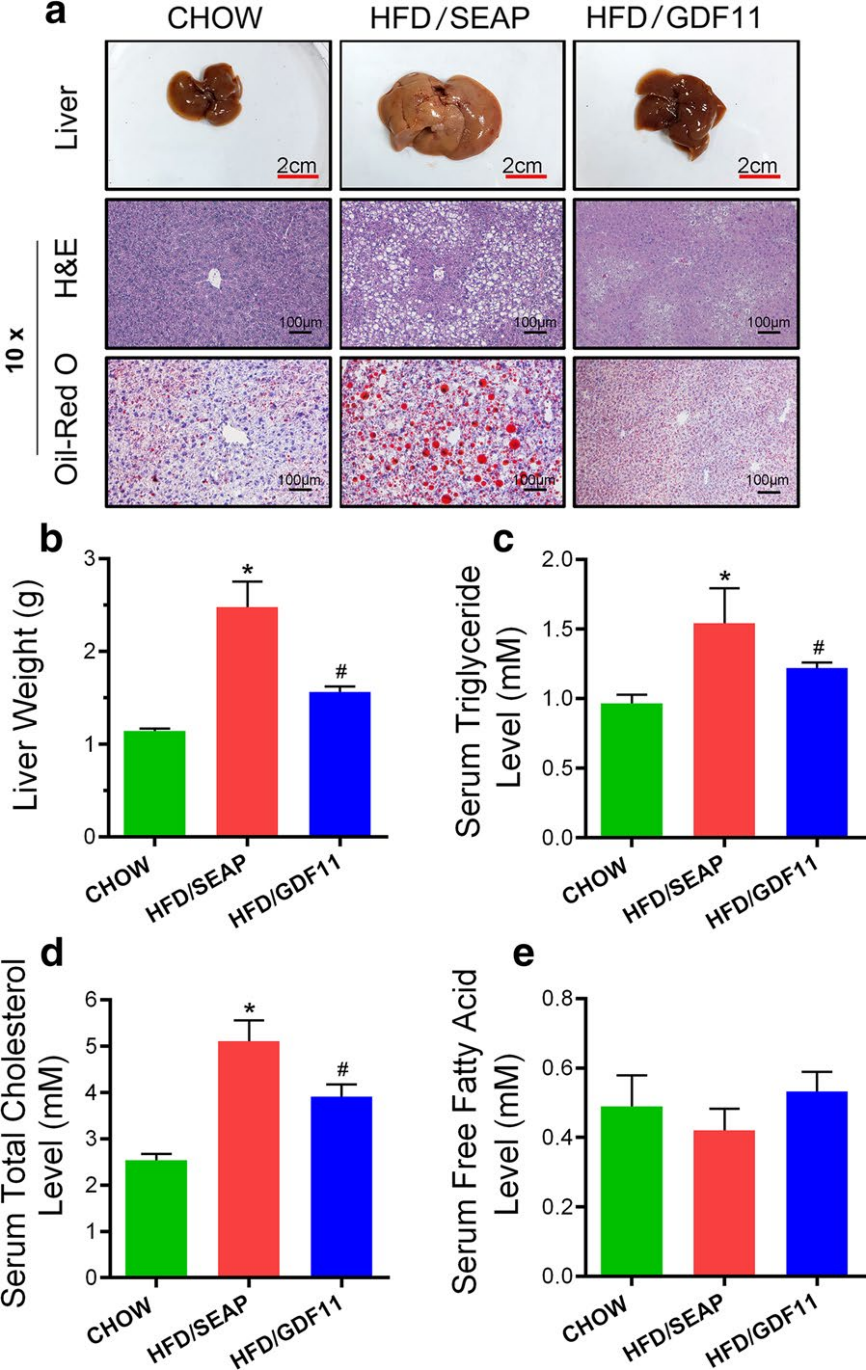
*Gdf11* gene transfer blocked HFD-induced fatty liver. Animals fed different diets and injected with different plasmids were euthanized at the end of the feeding period, liver samples were collected and analyses were performed. **a** Representative images of mouse livers, and histological presentations of the liver sections; **b** liver weight; **c** serum concentrations of triglyceride; **d** serum cholesterol level; and **e** serum free fatty acid level. The scale bars in H&E staining represent 100 µm. The scale bars in photo of liver represent 2 cm. Data represent mean ± SEM (n = 5). *P < 0.05 comparing to mice fed a Chow diet; ^#^P < 0.05 comparing to mice fed an HFD and injected with pLIVE-SEAP plasmids

Obesity is usually associated with excessive fat accumulation in the liver [25]. Results in Fig. 4a, b showed that *Gdf11* gene transfer blocked HFD-induced fat accumulation in the liver. *Gdf11* gene transfer resulted in a smaller liver comparing to that of HFD-fed control (Fig. 4a, b). H&E and Oil Red O staining showed that pLIVE-GDF11 treated mice had less and smaller lipid droplets and vacuoles in the liver comparing to HFD-fed control mice (Fig. 4a). The serum concentration of triacylglycerol and total cholesterol in GDF11-treated mice were significantly lower than HFD-fed control (Fig. 4c, d). Serum free fatty acid levels are similar among 3 animal groups (Fig. 4e). These results demonstrate that *Gdf11* gene transfer prevented HFD-induced fatty liver development.

### GDF11 improves metabolic homeostasis in obese mice

**Fig. 5:**
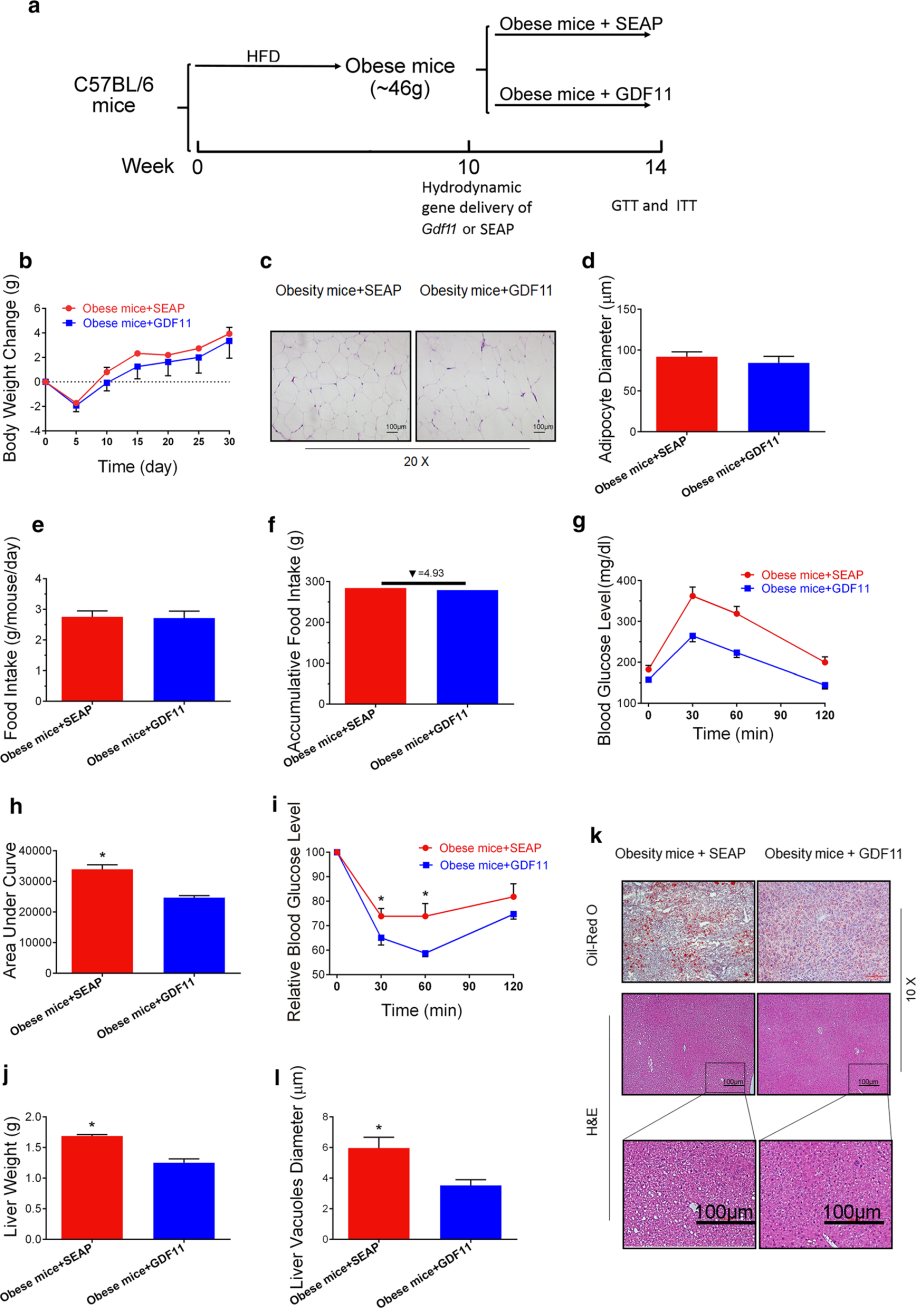
Effects of *Gdf11* gene transfer on high fat diet-induced obese mice. HFD-induced obese mice were injected with pLIVE-GDF11 or control plasmids and continued on HFD for 4 weeks. **a** Experiment schedule; **b** change of body weight after gene transfer; **c** histological images of EWAT (20 ×); **d** average diameter of adipocytes; **e** daily food intake; **f** accumulative food intake; **g** serum glucose level in GTT on day 23 after gene transfer; **h** area under the curve of GTT; **i** relative serum glucose concentrations in ITT on day 25 after gene transfer; **j** liver weight; **k** histological presentations of the liver sections (×10); **l** average diameter of vacuoles in hepatocytes in liver sections. Scale bars represent 100 µm. Each data point represents mean ± SEM (n = 5). *P < 0.05 comparing to mice injected with control plasmids

Beneficial effects of *Gdf11* gene transfer in preventing HFD-induced obesity and metabolic disorders such as glycemia, insulin resistance, and fatty liver have prompted us to explore its effects on animals with obesity. Toward this end, mice fed an HFD for 12 weeks (similar body weight around 44–48 g) were hydrodynamically injected with 25 µg of pLIVE-GDF11 or control plasmids (Fig. 5a). *Gdf11* gene transfer did not induce significant loss of body weight of the obese mice (Fig. 5b). H&E staining was performed on WAT on all animals (Fig. 5c). The average size of white adipocytes in animals with *Gdf11* overexpression was similar to that of obese control animals (Fig. 5d). There was no significant difference in food intake between pLIVE-GDF11 and pLIVE-SEAP treated groups (Fig. 5e, f). GTT and ITT were performed on these animals 4 weeks after gene transfer. GTT assay showed that animals with *Gdf11* gene transfer exhibited a higher glucose clearance rate (Fig. 5g). The AUC of GTT confirmed the improvement of glucose tolerance in GDF11 treated mice (Fig. 5h). ITT assay also showed an improvement of insulin sensitivity in mice treated with GDF11 (Fig. 5i). *Gdf11* gene transfer reduced HFD-induced fat accumulation in the liver (Fig. 5j–l). *Gdf11* gene transfer resulted in a lower liver weight comparing to that of obese control mice (Fig. 5j). H&E and Oil Red O staining showed that GDF11-treated mice had less and smaller lipid droplets and vacuoles in liver sections comparing to obese control mice (Fig. 5k, l). These results demonstrate that GDF11 ameliorates glucose intolerance and insulin resistance, improves glucose homeostasis, and reduces hepatic steatosis of obese mice.

### Impact of *Gdf11* gene transfer on STZ-induced type 2 diabetic mice

**Fig. 6:**
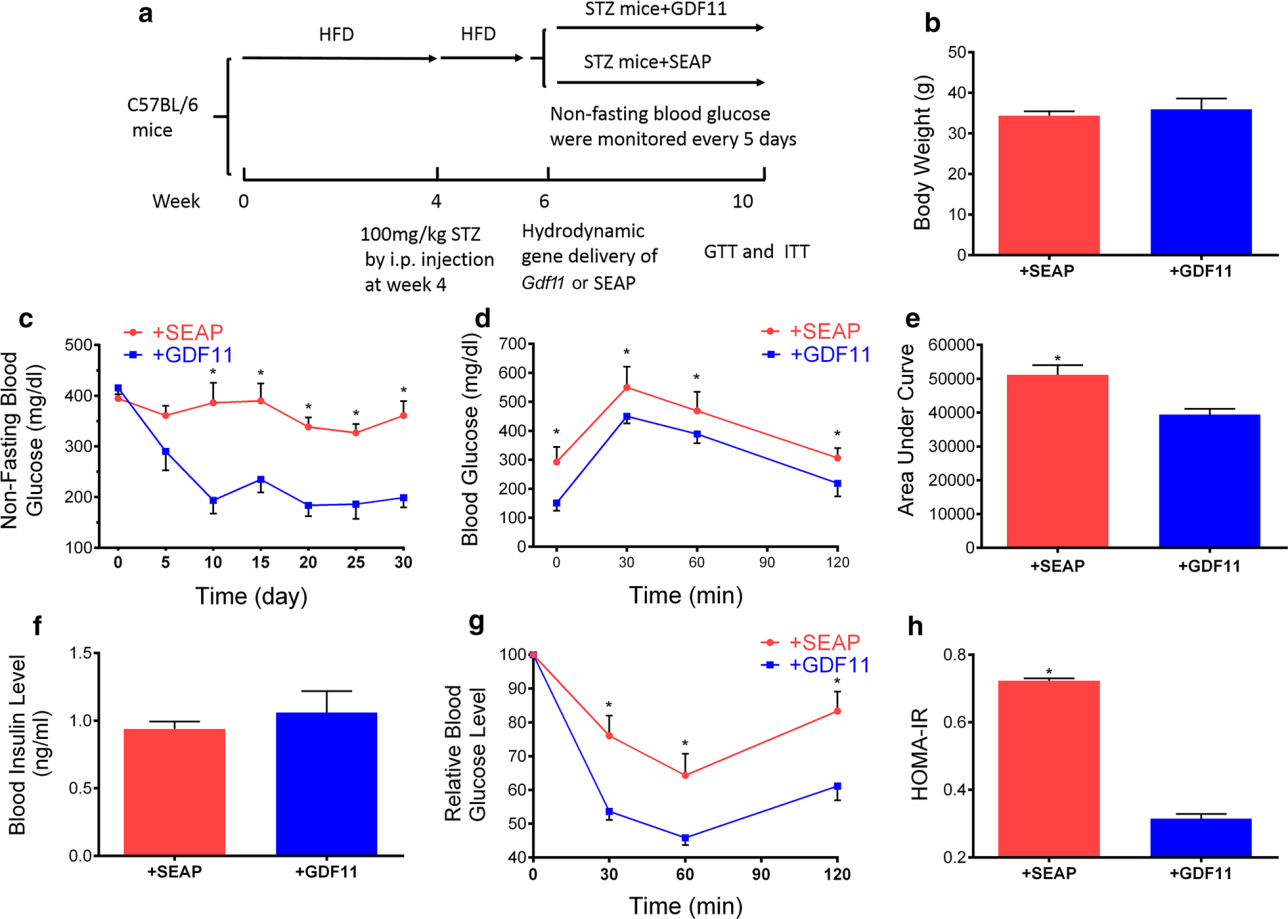
Effects of *Gdf11* gene transfer on STZ-induced type 2 diabetic mice. **a** Experiment schedule; **b** the body weight of mice 4 weeks after GDF11 treatment; **c** non-fasting blood glucose level after gene transfer; **d** serum glucose level in GTT; **e** area under the curve of GTT; **f** blood insulin level at the end of experiment; **g** serum glucose level in ITT; **h** results of HOMA-IR analysis for insulin resistance. Each data point represents mean ± SEM (n = 5). *P < 0.05 comparing to mice injected with pLIVE-SEAP

Similar study was conducted on mice with STZ-induced type 2 diabetes following the protocol shown in Fig. 6a. The body weight of these two group mice was similar 4 weeks after treatment (Fig. 6b). No significant difference in food intake was observed in these two group mice. Results in Fig. 6c showed that *Gdf11* gene transfer decreased the non-fasting blood glucose level and kept at a low level for more than 1 month. GTT assay demonstrated that *Gdf11* gene transfer improved glucose tolerance (Fig. 6d, e). No significant difference was seen in blood insulin levels between GDF11 treated and control mice (Fig. 6f). ITT assay showed a higher response to injected insulin, and HOMA-IR showed a decrease in insulin resistance in GDF11 treated mice (Fig. 6g, h). These results demonstrate that hydrodynamic injection of pLIVE-GDF11 plasmids had an anti-diabetic effect and improved glucose metabolism in STZ-induced type 2 diabetic mice.

### GDF11 increased oxygen consumption and energy expenditure

**Fig. 7:**
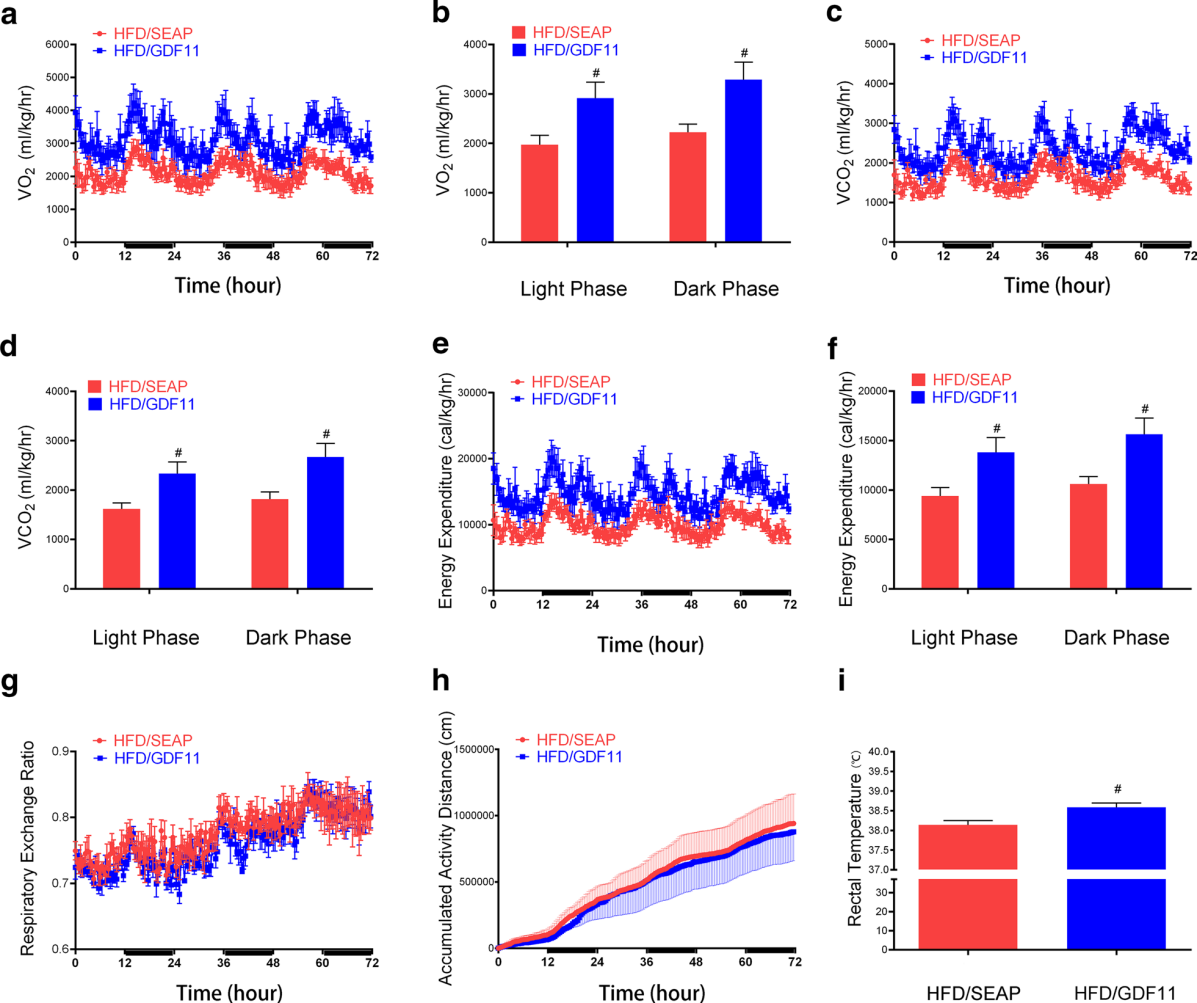
Metabolic cage study of animals with plasmid injection of pLIVE-GDF11 or pLIVE-SEAP. Mice were fed an HFD for 8 weeks after gene transfer and individually caged. Oxygen consumption and CO_2_ release by each mouse were monitored. The dark and light phases were indicated by black and white bars on the x-axis. **a** Oxygen consumption; **b** Average oxygen consumption in day and night; **c** CO_2_ output; **d** average CO_2_ output in day and night; **e** calculated energy expenditure; **f** average energy expenditure in day and night; **g** respiratory exchange ratio (RER); **h** accumulated activity; **i** the average rectal temperature after treatment. The rectal temperature of mice was measured weekly after hydrodynamic gene delivery. Data represent mean ± SEM (n = 5), ^#^P < 0.05

In order to investigate how GDF11 affected the body weight gain in HFD feeding mice, we conducted a series of measurements on animals housed in a metabolic cage. The oxygen consumption (VO_2_ and VCO_2_) was increased in GDF11 treated mice compared with HFD-fed control animals (Fig. 7a–d). The energy expenditure was upregulated after GDF11 treatment (Fig. 7e, f). The respiratory exchange ratio and activity were similar between GDF11 treated mice and HFD-fed control animals (Fig. 7g, h). The rectal temperature was tested weekly after gene transfer. The average rectal temperature also a little bit higher (~ 0.4 °C) in GDF11-treated mice than HFD-fed control and Chow-fed animals (Fig. 7i). These results demonstrate that *Gdf11* gene transfer elevates oxidation and energy expenditure in HFD-fed mice.

### GDF11 suppressed the inflammatory genes in WAT and elevated expression of thermogenesis genes in BAT

**Fig. 8:**
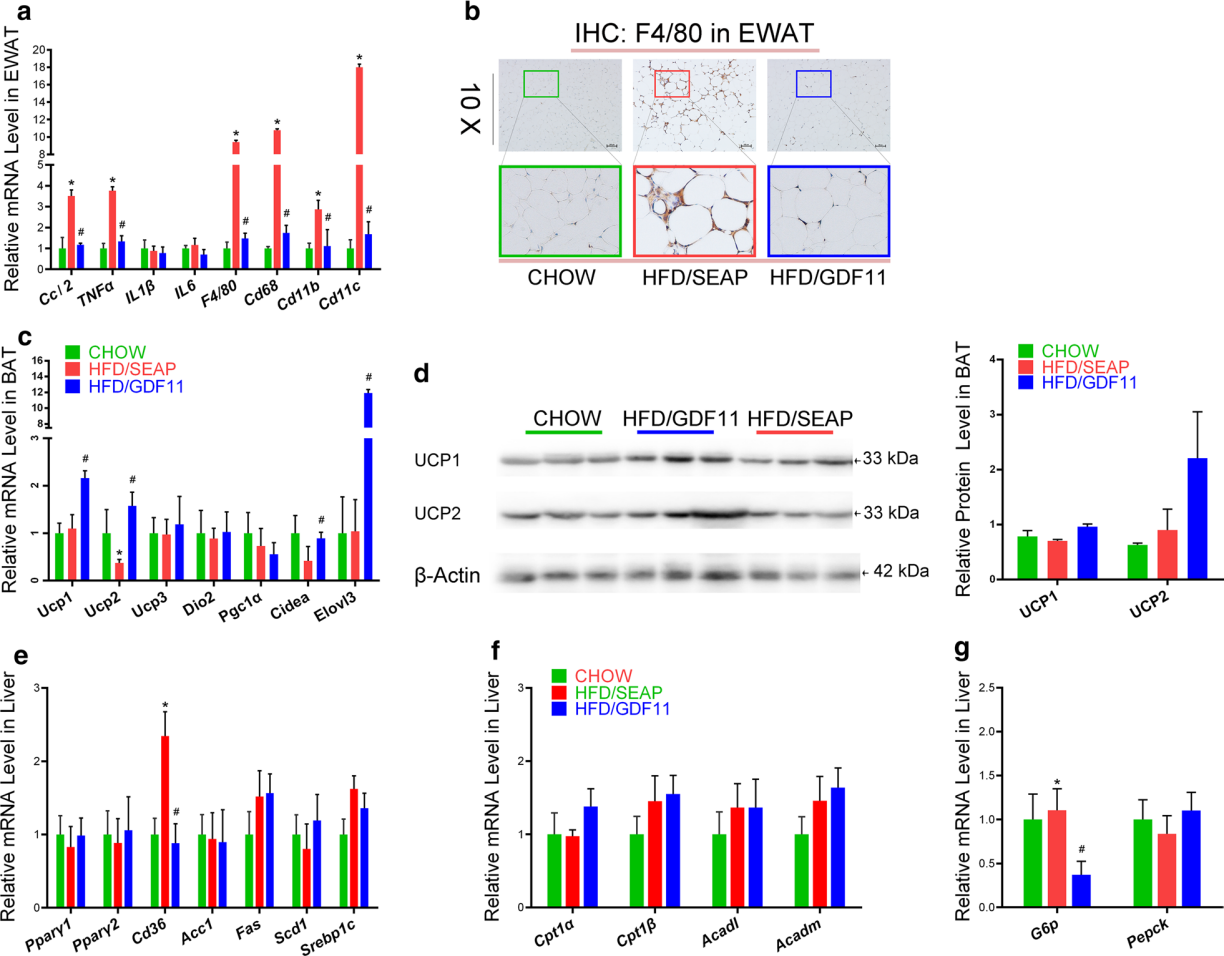
Impacts of *Gdf11* gene transfer on expression of genes involved in inflammation, thermogenesis, and glucose and lipid metabolism. Tissue was isolated from mice at the end of 8-week feeding period after gene transfer. **a** Relative mRNA levels of inflammatory genes and macrophage markers in the EWAT; **b** histoimmunochemistry of WAT with F4/80 antibody; **c** relative mRNA levels of thermogenic genes in brown adipose tissue; **d** the protein level of UCP1 and UCP2 in BAT (lift side) and scanning density of the protein bands (right side); **e** relative mRNA levels of genes involved in lipid metabolism in the liver; **f** relative mRNA levels of genes involved in β oxidation in the liver; **g** relative mRNA levels of genes involved in glucose metabolism in the liver. Each data point represents mean ± SEM (n = 5). *P < 0.05 comparing to mice fed regular Chow, ^#^P < 0.05 comparing to mice fed an HFD and injected with control plasmids

To investigate the underlying mechanisms of GDF11 in preventing HFD-induced obesity and obesity-associated complications, the mRNA levels of inflammatory genes were examined in WAT. HFD feeding markedly increased the expression of *Ccl2*, *Tnfα*, *F4/80*, *Cd68*, *Cd11b*, and *Cd11c* genes (Fig. 8a). *Gdf11* gene transfer significantly suppressed the expression of these genes in HFD-fed mice. The crown-like structures, an indication of macrophage infiltration into WAT, were labeled by F4/80 antibody in EWAT of mice. Significant less of F4/80 labeled crown-like structures were observed in GDF11-treated mice compared with HFD-fed control animals (Fig. 8b). These data indicate that GDF11 prevented HFD-induced inflammation and macrophage infiltration.

GDF11 treatment also significantly upregulated the mRNA expression of *Elovl3*, *Ucp1*, *Ucp2*, and *Cidea* in BAT comparing to that of HFD-fed control animals (Fig. 8c). The protein level of Ucp1 and Ucp2 in BAT was also higher in GDF11 treated mice compared with HFD-fed control group (Fig. 8d). These results suggest that GDF11 increased thermogenesis.

### GDF11 altered the expression of genes involved in glucose and lipid metabolism in the liver

To investigate the underlying mechanisms of GDF11 activity in preventing obesity and obesity-related complications, we also assessed the mRNA level of selected genes involved in glucose and lipid metabolism in the liver. *Gdf11* gene transfer significantly suppressed the expression of fatty acid translocase gene (*Cd36)* compared to that in HFD-fed control animals (Fig. 8e). No significant differences were seen in mRNA levels of *Pparγ1, Pparγ2, Acc1, Fas, Scd1,* and *Srebp1c* between animals with or without *Gdf11* gene transfer (Fig. 8e). GDF11 treatment also significantly reduced the expression of gluconeogenesis gene *G6p* comparing to that of the HFD-fed group but did not alter the expression of gluconeogenesis gene *Pepck* (Fig. 8g). Additionally, GDF11 treatment did not change the expression of genes involved in fatty acid β oxidation including *Cpt1α, Cpt1 β, Acadl*, and *Acadm* in GDF11 treated mice (Fig. 8f).

### GDF11 activated TGF-β/Smad2, AMPK, and PI3K/AKT/FoxO1 signaling pathways

**Fig. 9:**
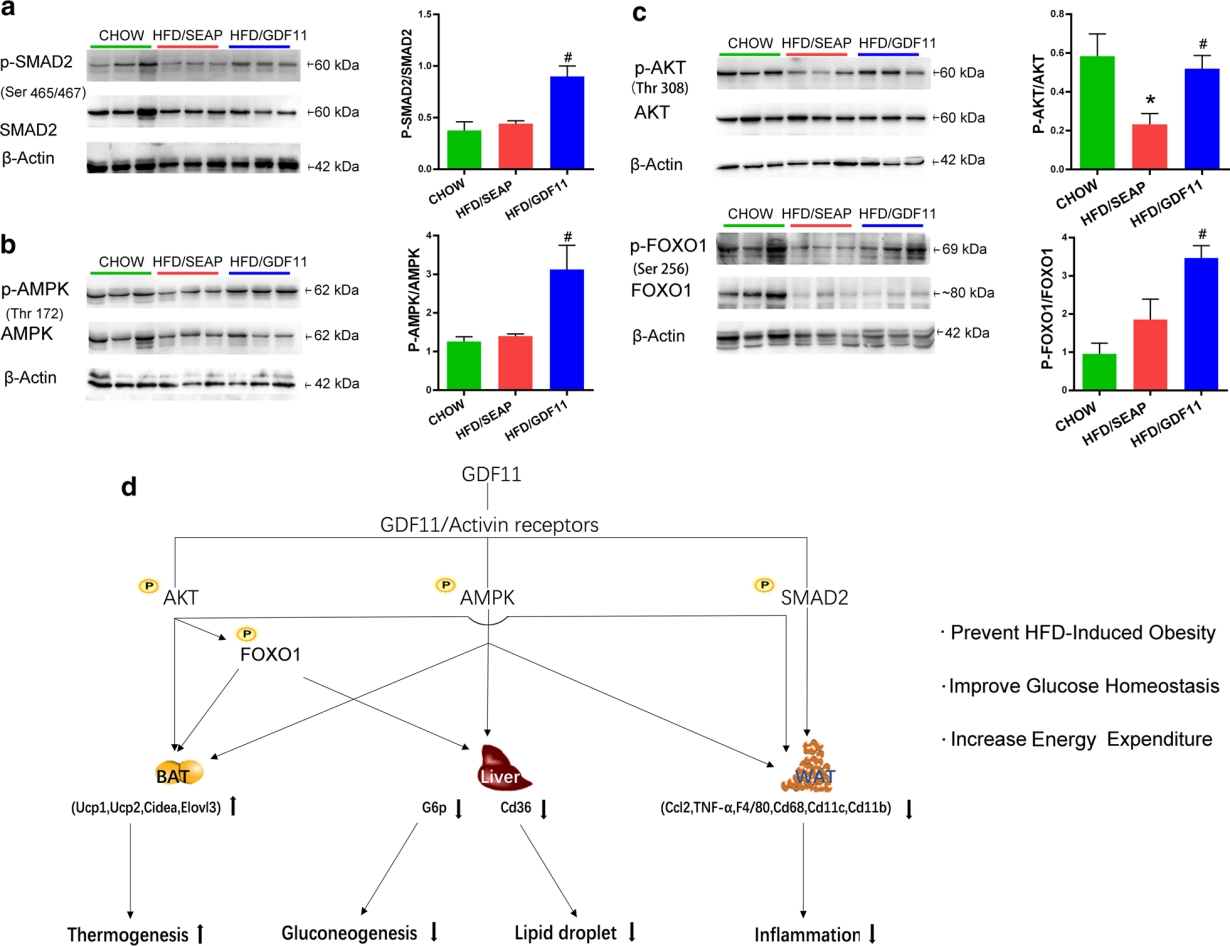
Impacts of Gdf11 gene transfer on TGF-β/Smad2, AMPK, and PI3K/AKT/FoxO1 signaling pathways. Total protein of EWAT was isolated from mice at the end of 8-week feeding period after gene transfer, and protein levels of selected genes were determined by western blotting in GDF11-treated, HFD-fed control and Chow control animals. **a** Western blotting analysis for SMAD2, p-SMAD2 and β-actin; **b** western blotting analysis for AMPK, p-AMPK, and β-actin; **c** western blotting analysis for AKT, p-AKT, FoxO1, p-FoxO1, and β-actin; **d** depicted pathways impacted by gene transfer of *Gdf11* in HFD-induced obesity mouse models. GDF11 treatment activates AMPK, AKT, and SMAD2 signal pathways, which increases the energy expenditure of mice, upregulates the expression of genes responsible for thermoregulation in brown adipose tissue, downregulates the expression of inflammatory genes in white adipose tissue and those involved in hepatic lipid and glucose metabolism. Each data point represents mean ± SEM, *P < 0.05 comparing to mice fed regular Chow, ^#^P < 0.05 comparing to mice fed an HFD and injected with control plasmids

To further investigate the possible signaling pathways by which GDF11 prevents obesity and obesity-related complications, western blotting was performed to examine the canonical and noncanonical signal cascades of GDF11 in WAT. GDF11 is a member of the TGF-β family, having the canonical TGF-β/Smad signaling cascade [1]. The phosphorylation level of Smad2 in white adipose tissue was significantly increased in GDF11 treated mice compared to that in HFD-fed control mice (Fig. 9a). No significant differences were observed in the phosphorylation level of Smad2 between HFD control and Chow groups. No significant differences were seen in total protein level of Smad2 between animals with or without GDF11 treatment. We further detected the noncanonical pathways possibly involved in GDF11 cascades. *Gdf11* gene transfer significantly increased AMPK phosphorylation in WAT compared to that in HFD-fed control mice (Fig. 9b), indicating that GDF11 activated the AMPK signaling pathways. In addition, *Gdf11* gene transfer significantly increased the phosphorylation level of AKT compared to that in HDF-fed control animals (Fig. 9c). The FoxO1 phosphorylation, a downstream event of AKT activation, was also significantly increased in the WAT of GDF11 treated mice compared to HFD-fed control (Fig. 9c), indicating that GDF11 activated the PI3K/AKT/FoxO1 pathway.

## Discussion

In this study, we demonstrate that hydrodynamic transfer of *Gdf11* gene prevented HFD induced obesity, hyperglycemia, insulin resistance and fatty liver in mice. *Gdf11* gene transfer also improved metabolic homeostasis in obese mice and mice with STZ-induced diabetes.

The finding that GDF11 is capable of improving glucose metabolism and metabolic homeostasis is consistent with the results of previous studies using *db/db* and STZ-induced diabetic mice as an animal model [15, 17]. Two mechanisms might be involved. First of all, it is possible that overexpression of GDF11 promoted β-cell differentiation and development of pancreas through Smad2 and PI3K/AKT/FoxO1 signal pathways [17, 26, 27] as previously shown that administration of rGDF11 protein or AAV-GDF11 vectors increased the survival of β-cells in diabetic mice [17]. Results in Fig. 9 confirmed that GDF11 activated TGF-β/Smad2 and PI3K/AKT/FoxO1 pathways in WAT. The second possibility is that GDF11 blocks pathways that lead to metabolic disorders that involve inflammatory cytokines and activation of macrophages [21, 22, 28]. It was previously shown that GDF11 has anti-inflammatory effects [15, 19, 29]. Treatment with rGDF11 attenuated inflammation in psoriasis-like skin inflammation mice and obese mice [15, 19]. rGDF11 or AAV-mediated *Gdf11* gene transfer reduced inflammatory cytokines and suppressed the expression of inflammatory genes in aortas, and increased anti-inflammation cytokine IL10 in *apoE*^−*/*−^ mice [15]. The conclusions of these earlier studies are supported by our results demonstrating that GDF11 suppressed the expression of inflammatory genes in WAT including *Tnfα* and *Ccl2* and macrophage marker genes such as *F4/80, Cd68, Cd11b,* and *Cd11c* in adipose tissue (Fig. 8a). GDF11 also reduced the development of crown-like structures in adipose tissue, a sign of macrophage infiltration and migration into WAT (Figs. 2c, 8b). As inflammation is known for its role in inducing obesity and metabolic complications, the anti-inflammation function of GDF11 is likely one of the reasons for the beneficial effects seen in animals employed in our study (Fig. 9d). In addition, GDF11-induced decrease in expression of gluconeogenesis gene *G6P* (Fig. 8g), the downstream of PI3K/AKT/FoxO1 signal pathway, in the liver, may also play critical role in reducing blood glucose level and reestablishing glucose homeostasis (Fig. 9d). Moreover, AMPK activity was induced by GDF11 (Fig. 9b), which may also play an important role in glucose uptake, and glucose homeostasis [30, 31].

The fact that no significant weight gain was seen in GDF11-treated animals while there was no difference in their food intake (Fig. 1f, g) and activity compared to the HFD-fed control animals suggests that GDF11-treated animals consume more energy than HFD-fed control animals, which was confirmed by metabolic cage assay that the oxygen consumption and energy expenditure were significantly increased after GDF11 treatment (Fig. 7). The higher level of energy consumption is likely achieved by converting food energy to heat which is released. The rectal temperature was a little bit higher in GDF11-treated mice comparing with HFD-fed control and Chow animals (Fig. 7i). GDF11-induced increase in Ucp1 and Ucp2 expression seen in BAT (Fig. 8c, d) supports the notion that heat release is the mechanism that prevents the animals with *Gdf11* gene transfer from gaining weight. Increased thermogenesis is also supported by elevated expression of *Elovl3* which is an important component for lipid recruitment in BAT [32]. *Elovl3* gene expression is significantly increased during cold stimulation linking *Elovl3* to the thermogenic process [33]. These suggest that GDF11-mediated thermogenesis and energy consumption play important roles in prevention of HFD-induced obesity and metabolic disorders.

Previous studies have demonstrated that GDF11 binds to activin type I/II receptors and activates the canonical TGF-β/Smad signaling pathway. TGF-β/Smad pathway was involved in improving islet β-cell function, glucose homeostasis, and lipid metabolism [17, 34–36]. In the current study, we also revealed that GDF11 significantly increased the phosphorylation of SMAD2 in WAT (Fig. 9a). In addition, GDF11 also increased the phosphorylation of AKT and FoxO1 (Fig. 9c) which play an important role in the PI3K/AKT/FoxO1 signal cascade critical for regulating lipid metabolism and glucose homeostasis in adipose tissue, liver, pancreas, and skeletal muscle [37, 38]. Moreover, the phosphorylation of AMPK was also upregulated by GDF11 (Fig. 9b). It is known that PI3K/AKT/FoxO1 and AMPK pathways, the noncanonical GDF11 signal cascades, have cross-talk with canonical TGF-β/Smad cascade of GDF11 influencing metabolic homeostasis [31, 39, 40]. These results suggest that GDF11 function in preventing metabolic disorders is achieved through TGF-β/Smad2, PI3K/AKT/FoxO1, and AMPK pathways.

Hydrodynamic injection of *Gdf11* gene generated the sustained blood circulating level of GDF11 and long-term anti-diabetic effects, indicating that GDF11 has therapeutic potential in treating diabetes and obesity-related metabolic diseases. However, the detailed mechanisms of GDF11 in preventing obesity and fatty liver remain elusive, as well as the mechanisms of GDF11 in regulating oxygen consumption, thermogenesis, and inflammation. Further studies are needed to investigate the mechanisms of GDF11 in regulating adipocyte development and metabolic homeostasis. Moreover, the hydrodynamic tail vein injection is a physical method of gene delivery to hepatocytes in the liver [41]. The overexpressed GDF11 was secreted into the blood circulation and reached multiple tissues. Additional work is needed to investigate the long term effect of GDF11 in bone, skeletal muscle, and other tissues where GDF11 activities have been demonstrated [16, 42, 43]. Differences reported in different animal models about GDF11 functions need additional investigation. For example, the role of GDF11 on the development of skeletal muscle was different in young and aged mice [44]. AAV-mediated *Gdf11* gene transfer blocks the growth and development of skeletal muscle in neonatal mice [42], but supplementary of GDF11 in old mice restored skeletal muscle dysfunctions, enhanced muscle fibers, and increased the number of multinucleated myotubes [4]. Controversial results have also been reported about the function of GDF11 in aging-related cardiac hypertrophy, cerebral vasculature dysfunctions, and cachexia [1, 15, 45, 46]. The effects of GDF11 on aging have been the focus of GDF11-related research in recent years, and future studies will surely shine light on these seeming controversy results.

## Conclusions

In summary, we demonstrate that *Gdf11* gene transfer alleviated HFD-induced obesity, hyperglycemia, insulin resistance and fatty liver development in mice. GDF11 also improves glucose homeostasis in obese mice and mice with STZ-induced diabetes. The mechanism study revealed that GDF11 upregulated energy expenditure, upregulated thermogenic genes in BAT, suppressed the expression of inflammatory genes in WAT, and decreased expression of genes responsible for lipid and glucose metabolism in the liver. The signal mechanism underlying the beneficial effects of GDF11 includes TGF-β/Smad2, PI3K/AKT/FoxO1, and AMPK pathways. GDF11 function demonstrated here would suggest a potential use of GDF11 by gene transfer for the treatment of metabolic diseases.

## Supplementary information

**Additional file 1: Table S1. Primer sequences for PCR analysis.**

**Additional file 2: Figure S1. Impacts of*Gdf11*gene transfer on Chow-fed mice.** Chow-fed mice were injected with pLIVE-GDF11 or control plasmids and continued on HFD for 6 weeks. (A) Change of body weight after gene transfer; (B) Daily food intake; (C) Fasting blood glucose level after gene transfer; (D) Serum glucose level in GTT 6 weeks after gene transfer.

## Data Availability

The datasets used and/or analysed during the current study are available from the corresponding author on reasonable request.

## Abbreviations

GDF11: growth differentiation factor 11
STZ: streptozotocin
HFD: high-fat diet
GTT: glucose tolerance test
ITT: insulin tolerance test
HOMA-IR: homeostatic model assessment for insulin resistance
AST: aspartate aminotransferase
ALT: alanine aminotransferase
WAT: white adipose tissue
EWAT: epididymal white adipose tissue
SWAT: subcutaneous white adipose tissue
IWAT: inguinal white adipose tissue
dlWAT: dorsolumbar subcutaneous white adipose tissue
asWAT: anterior subcutaneous white adipose tissue
BAT: brown adipose tissue
AUC: area under the curve
