# Supplementary material for: Gdf11 gene transfer prevents high fat diet-induced obesity and improves metabolic homeostasis in obese and STZ-induced diabetic mice
Source: J Transl Med. 2019 Dec 17;17:422. doi: 10.1186/s12967-019-02166-1 (PMC6915940; doi:10.1186/s12967-019-02166-1)
Supplement: Supplementary file 1 — Additional file 1: Table S1. Primer sequences for PCR analysis. [file 12967_2019_2166_MOESM1_ESM.docx]

**Table S1. Primer sequences for PCR analysis**

| **Gene** | **Forward primer sequence** | **Reverse primer sequence** |
| --- | --- | --- |
| *Pparγ1* | GGAAGACCACTCGCATTCCTT | GTAATCAGCAACCATTGGGTCA |
| *Pparγ2* | TCGCTGATGCACTGCCTATG | GAGAGGTCCACAGAGCTGATT |
| *Cd36* | CCTTAAAGGAATCCCCGTGT | TGCATTTGCCAATGTCTAGC |
| *Acc1* | GCCTCTTCCTGACAAACGAG | TGACTGCCGAAACATCTCTG |
| *Fas* | AGAGATCCCGAGACGCTTCT | GCCTGGTAGGCATTCTGTAGT |
| *Scd1* | TTCTTACACGACCACCACCA | CCGAAGAGGCAGGTGTAGAG |
| *Srebp1c* | CCCTGTGTGTACTGGCCTTT | TTGCGATGTCTCCAGAAGTG |
| *G6p* | CGACTCGCTATCTCCAAGTGA | GTTGAACCAGTCTCCGACCA |
| *Pepck* | AAGCATTCAACGCCAGGTTC | GGGCGAGTCTGTCAGTTCAAT |
| *Cpt1α* | CTCCGCCTGAGCCATGAAG | CACCAGTGATGATGCCATTCT |
| *Cpt1β* | GGTCTCTTCTTCAAGGTCTG | CGAGGATTCTCTGGAACTGC |
| *Acadl* | TCTTTTCCTCGGAGCATGACA | GACCTCTCTACTCACTTCTCCAG |
| *Acadm* | GGGTTTAGTTTTGAGTTGACGG | CCCCGCTTTTGTCATATTCCG |
| *Ccl2* | CTGGATCGGAACCAAATGAG | CGGGTCAACTTCACATTCAA |
| *Tnfα* | CCCTCACACTCAGATCATCTTCT | GCTACGACGTGGGCTACAG |
| *IL1β* | GCAACTGTTCCTGAACTCAACT | ATCTTTTGGGGTCCGTCAACT |
| *IL6* | TAGTCCTTCCTACCCCAATTTCC | TTGGTCCTTAGCCACTCCTTC |
| *F4/80* | CTTTGGCTATGGGCTTCCAGTC | GCAAGGAGGACAGAGTTTATCGTG |
| *Cd68* | CCATCCTTCACGATGACACCT | GGCAGGGTTATGAGTGACAGTT |
| *Cd11b* | ATGGACGCTGATGGCAATACC | TCCCCATTCACGTCTCCCA |
| *Cd11c* | CTGGATAGCCTTTCTTCTGCTG | GCACACTGTGTCCGAACTC |
| *Ucp1* | AGGCTTCCAGTACCATTAGGT | CTGAGTGAGGCAAAGCTGATTT |
| *Ucp2* | ATGGTTGGTTTCAAGGCCACA | CGGTATCCAGAGGGAAAGTGAT |
| *Ucp3* | CTGCACCGCCAGATGAGTTT | ATCATGGCTTGAAATCGGACC |
| *Dio2* | AATTATGCCTCGGAGAAGACCG | GGCAGTTGCCTAGTGAAAGGT |
| *Pgc1α* | TATGGAGTGACATAGAGTGTGC | CCACTTCA ATCCACCCAGAAAG |
| *Cidea* | TGACATTCATGGGATTGCAGAC | GGCCAGTTGTGATGACTAAGAC |
| *Elovl3* | TTCTCACGCGGGTTAAAAATGG | GAGCAACAGATAGACGACCAC |
| *Gapdh* | AGGTCGGTGTGAACGGATTTG | TGTAGACCATGTAGTTGAGGTCA |
| *mGdf11* | TATGGATCCATGGTGCTCGCGGCCCCGCT | TACGAGCTCTTAGGAGCAGCCACATCG |
