# Supplementary material for: Gdf11 gene transfer prevents high fat diet-induced obesity and improves metabolic homeostasis in obese and STZ-induced diabetic mice
Source: J Transl Med. 2019 Dec 17;17:422. doi: 10.1186/s12967-019-02166-1 (PMC6915940; doi:10.1186/s12967-019-02166-1)
Supplement: Supplementary file 2 — Additional file 2: Figure S1. Impacts ofGdf11gene transfer on Chow-fed mice. Chow-fed mice were injected with pLIVE-GDF11 or control plasmids and continued on HFD for 6 weeks. (A) Change of body weight after gene transfer; (B) Daily food intake; (C) Fasting blood glucose level after gene transfer; (D) Serum glucose level in GTT 6 weeks after gene transfer. [file 12967_2019_2166_MOESM2_ESM.pdf]

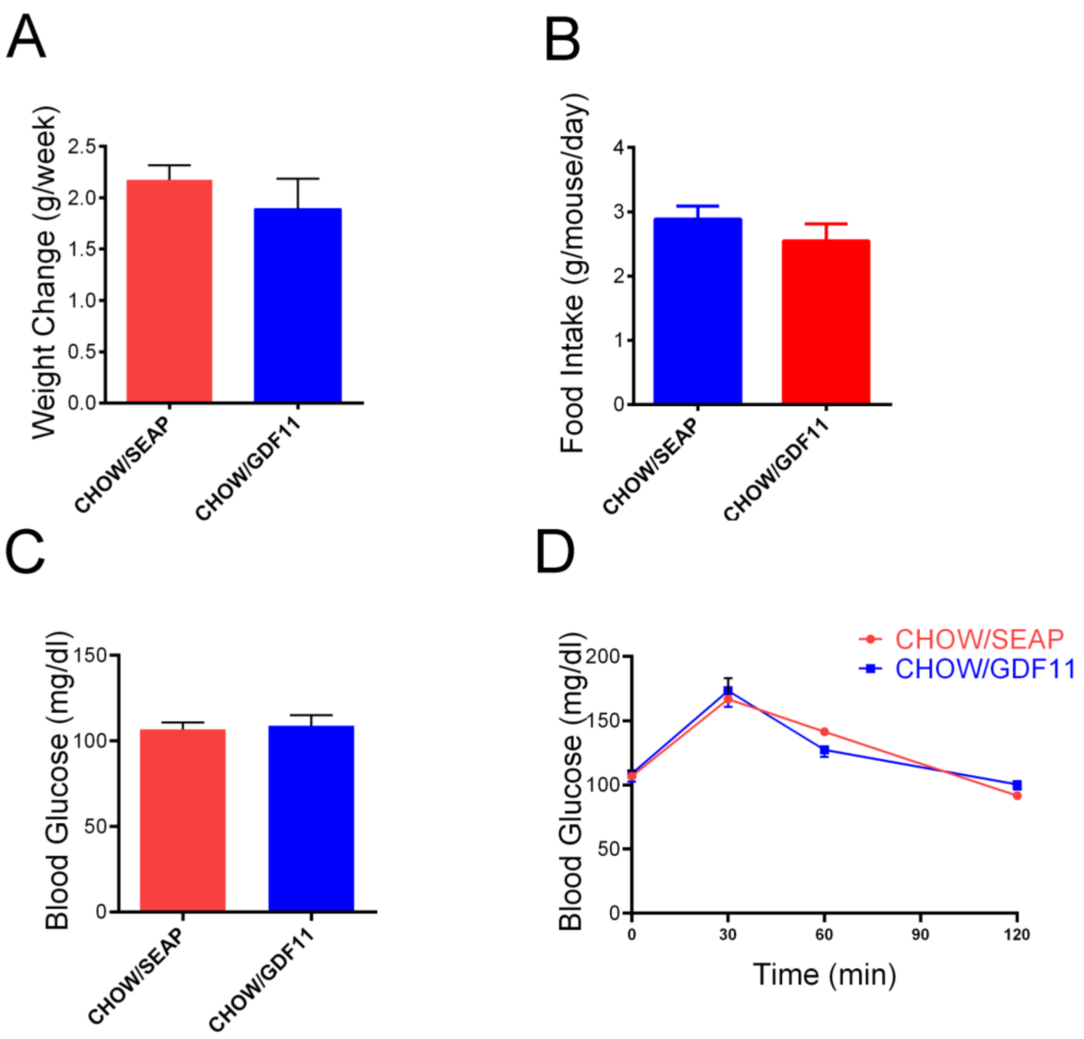

Figure S1. Impacts of *Gdf11* gene transfer on Chow-fed mice. Chow-fed mice were injected with pLIVE-GDF11 or control plasmids and continued on HFD for 6 weeks. (A) Change of body weight after gene transfer; (B) Daily food intake; (C) Fasting blood glucose level after gene transfer; (D) Serum glucose level in GTT on day 6 weeks after gene transfer.
